# Semi-Automated Glycoproteomic Data Analysis of LC-MS Data Using GlycopeptideGraphMS in Process Development of Monoclonal Antibody Biologics

**DOI:** 10.3389/fchem.2021.661406

**Published:** 2021-05-18

**Authors:** Kuin Tian Pang, Shi Jie Tay, Corrine Wan, Ian Walsh, Matthew S. F. Choo, Yuan Sheng Yang, Andre Choo, Ying Swan Ho, Terry Nguyen-Khuong

**Affiliations:** Bioprocessing Technology Institute, Agency for Science Technology and Research (A*STAR), Queenstown, Singapore

**Keywords:** GraphMS, BioAccord, IgG, trastuzumab, adalimumab, glycoproteomic, process development, biologics

## Abstract

The glycosylation of antibody-based proteins is vital in translating the right therapeutic outcomes of the patient. Despite this, significant infrastructure is required to analyse biologic glycosylation in various unit operations from biologic development, process development to QA/QC in bio-manufacturing. Simplified mass spectrometers offer ease of operation as well as the portability of method development across various operations. Furthermore, data analysis would need to have a degree of automation to relay information back to the manufacturing line. We set out to investigate the applicability of using a semiautomated data analysis workflow to investigate glycosylation in different biologic development test cases. The workflow involves data acquisition using a BioAccord LC-MS system with a data-analytical tool called GlycopeptideGraphMS along with Progenesis QI to semi-automate glycoproteomic characterisation and quantitation with a LC-MS1 dataset of a glycopeptides and peptides. Data analysis which involved identifying glycopeptides and their quantitative glycosylation was performed in 30 min with minimal user intervention. To demonstrate the effectiveness of the antibody and biologic glycopeptide assignment in various scenarios akin to biologic development activities, we demonstrate the effectiveness in the filtering of IgG1 and IgG2 subclasses from human serum IgG as well as innovator drugs trastuzumab and adalimumab and glycoforms by virtue of their glycosylation pattern. We demonstrate a high correlation between conventional released glycan analysis with fluorescent tagging and glycopeptide assignment derived from GraphMS. GraphMS workflow was then used to monitor the glycoform of our in-house trastuzumab biosimilar produced in fed-batch cultures. The demonstrated utility of GraphMS to semi-automate quantitation and qualitative identification of glycopeptides proves to be an easy data analysis method that can complement emerging multi-attribute monitoring (MAM) analytical toolsets in bioprocess environments.

## Introduction

Biologic glycosylation impacts efficacy, function, and clinical outcomes of the molecule. For example, with antibody drugs, the fucosylation or galactosylation heavily impacts the antibody cytotoxicity or complement-dependent cytotoxicity respectively (Shields et al., 2002; Hodoniczky et al., 2005). There is an increase rate of new approved monoclonal antibody (mAb) therapeutic products from different IgG subclasses (Kaplon and Reichert, 2021). Each IgG subclass can exhibit different degrees of glycosylation (Lippold et al., 2020). Ensuring the correct glycosylation of the product is therefore a major challenge within bioprocessing operations. While glycosylation can be altered significantly *via* cell-line engineering (Goh et al., 2014), there is still much to learn about how glycosylation can be manipulated using different cultured conditions during cell fermentation (Walsh et al., 2020). For example, there have been studies that demonstrated that dissolved oxygen tension, pH, NH_4_^+^, temperature and nutrient supplementation during upstream culturing can affect the glycan profile of mAb (Zhang et al., 2016). Additionally, Schiestl et al. (2011) analysed different lots of Rituxan® and found that differences in the abundance of afucosylated G0 gylcan in different batches had an impact of antibody-dependent cellular cytotoxicity (ADCC); the difference in fucosylation was attributed to batch-to-batch variability in the manufacturing process (Schiestl et al., 2011). These challenges during lead development, or clone selection activities, highlight the importance to relay information such as degree of glycosylation in an expedient manner to ensure an efficient decision-making process.

To that end, the availability of a robust and sensitive glyco-analytical platform such as that centered around liquid chromatography-mass spectrometry (LC-MS) are being implemented to facilitate detailed understanding of glycosylation and bioprocessing operation (Tsai and Chen, 2017). With regards to mass spectrometry (MS), intact glycans/glycopeptides can be conventionally identified via their intact mass in MS and the nature of their fragmentation in MS/MS mode. Measuring a glycan/glycopeptides intact mass (MS) can often be enough to elucidate the composition of the molecule of interest. Observing the fragmentation of intact molecules (MS/MS) is often straightforward to elucidating the linear sequence of molecules such as proteins and oligonucleotides. With respect to fragmentation of glycans, the resultant isobaric masses, only confirm the composition of the glycan (in positive mode) and do little in the way of offering information such as their branching, topology and isomerism without significant effort with advanced glycomic techniques (Zhou et al., 2017). Recent additions to configurations of mass spectrometers mean that other layers of information with which to help characterise the glycoconjugates. Electron transfer dissociation (ETD), for example, has been used effectively to identify the site of the glycan on the glycopeptide (Riley and Coon, 2018). Additionally, ion mobility has recently been used to characterise glycan arm isomers (Pallister et al., 2020). As one can appreciate, significant expertise and effort as well as advanced mass specrometers with higher specifications are often needed to resolve more information about a glyco-conjugates’ structure.

The complexity of LC-MS systems for glycopeptide analysis has necessitated the use of simplified mass spectrometers and analytical workflows capable of performing analyses at intact sub-unit, peptide and released glycan level with optimised compliance-ready workflows (Rogers et al., 2018). These workflows are known as multi-attribute monitoring (MAM) and they can be performed with mass spectrometers and have been used to identify biotherapeutic attributes such as deamidation, oxidation, pyroglutamate formation, lysine clipping, aspartate isomerization and glycosylation in one analysis (Song et al., 2021). In most instances, the accurate mass of the peptides and PTMs are compared and validated by cross-referencing these measurements to a curated database. MAM is being increasingly adopted within the biopharmaceutical industry because in principle the analytical workflows which include sample preparation and data processing techniques and instrumentation are robust enough to be harmonized across the biologic development value chain and across different manufacturing sites (Song et al., 2021). This harmonization decreases the cost of assay development; however, there is the potential challenge that these techniques can miss unexpected PTMs such as glycosylation which may not be described in the analytical databases. Therefore, a database –independent approach would be ideal to identify and profile this unexpected glycosylation.

LC-MS technologies generate large volumes of data and data analysis approaches are the largest bottleneck to relaying glycosylation information in a timely manner. Several approaches to decipher or process information from these configurations are quite involved and there exist numerous efforts to facilitate this. Extensive reviews of the software and informatics resources to help with glycan analysis can be found here (Hu et al., 2016; Walsh et al., 2016; Campbell, 2017; Abrahams et al., 2020). In principle, data analysis for MS based glycoproteomic can be summarized into a few strategies; one of which is the accurate mass measurement of the glycopeptide using MS data and deduction of the glycan composition based on the MS/MS fragments. These solutions often require matching measured masses and their fragments to a curated or simulated glycan and peptide database for confirmation. These approaches therefore make it difficult to identify unexpected glycan compositions that may not be described in databases. Furthermore, they often depend quite heavily upon human intervention and interpretation of the results. Recently, informatic approaches such as the application of GlycopeptideGraphMS (GraphMS) have exploited patterns in glycopeptide detection in a LC-MS to analyse glycopeptides (Choo et al., 2019). GraphMS is a graph theoretical algorithm that groups LC-MS features into grouped networks that pertain to N-glycopeptides sharing the same peptide backbone. Doing so offers higher confidence in characterisation and validation of glycopeptides in any complex sample. It has previously been exploited to comprehensively characterise AXL protein, a glycoprotein with higher complex glycosylation heterogeneity compared to antibody molecules, and IgG (Choo et al., 2019; Lippold et al., 2020).

In this study, we explore the utility of GraphMS on data generated from a BioAccord LC-MS system—a system that is simple to operate for LC-MS expert and non-expert alike without compromising the quality, resolution and accuracy of data—in several use cases that represent the value chain of a bioprocessing operation that produces the IgG biosimilar trastuzumab (anti-HER-2). We intend on using a workflow that involves collecting data from the instrument, deconvolution through Progenesis QI and subsequent glycopeptide mapping with GraphMS to characterise the glycopeptides and their compositions qualitatively and quantitatively. This approach is robust, is database-independent and requires very little user intervention other than to confirm the mass of reference nodes. We apply this approach to different biologic development scenarios and compare this technique to conventional fluorescently labeled released glycan analysis protocols.

## Materials and Methods

### Chemicals, Materials and Reagents

Serum IgG was purchased from Sigma-Aldrich (United States). Trastuzumab (Herceptin), and adalimumab (Humira) were purchased from Roche (Switzerland), and Abbott (United States), respectively. Unless otherwise stated, all chemicals and reagents were purchased from Sigma-Aldrich (United States).

### Production and Isolation of In-House Produced Trastuzumab Biosimilar

Trastuzumab biosimilar was produced in CHO-K1 cells cultured in 14-days fed-batch cultures using Ambr250 bioreactors. Each fed-batch culture was started by inoculation of cells into 200 ml of EX-CELL Advanced CHO Fed-batch medium (SAFC media, Sigma Aldrich, United States) or HyClone ActiPro (GE media, GE Healthcare, United States) cell culture media supplemented with 6 mM of glutamine but without MTX. The inoculated viable cell density was 3 × 10^5^ cells/ml. The cultures were maintained at 37°C. Dissolved oxygen was maintained at 50%, while pH was maintained at 7.1. The cultures were mixed using dual pitch blade impellers stirring at 300 rpm. For cultures in EX-CELL Advanced CHO Fed-batch medium, 10% of EX-CELL Advanced CHO Feed 1 (with glucose) were fed into culture at day 3, 5, 7, 9 and 14. For cultures in HyClone ActiPro media, 6% Cell boost 7a and 0.6% Cell boost 7b were fed into culture at day 3, 5, 7, 9 and 14. Glucose concentration in the media was analysed on days 3, 5, 7, 9 and 14 *via* the Nova BioProfile 100 plus bioanalyser (Nova Biomedical, United States). When the concentration of glucose dropped to below 2 g/L, a specified volume of 45% glucose stock was added into fed-batch cultures in order to achieve final glucose concentration at 6 g/L. Media samples were taken at days 3, 5, 7, 9 and 14 for glycosylation analysis. Briefly, the media samples were filtered using a 0.45 μm membrane filter (MerckMillipore, United States) and concentrated using Amicon Ultracel-10k centrifugal filter units (MerckMillipore, United States) before trastuzumab biosimilar was purified using Protein A HP SpinTrap column (GE Healthcare, United States). Purified trastuzumab biosimilar was buffer-exchanged into water using Amicon Ultracel-10k centrifugal filter units to eliminate any salts and nucleophiles prior to drying them using a CentriVap centrifugal vacuum concentrator (Labconco, United States).

### Proteolytic Digestion of Antibodies

Proteolytic digestion of antibody was performed using the modified single-pot, solid-phase-enhanced sample preparation (SP3) technology as described previously (Hughes et al., 2019). Briefly, 20 μg of dried antibody was denatured in 15 μL of 8 M Urea (dissolved in 50 mM ammonium bicarbonate), followed by 30 min incubation at 60°C with 10 μL of dithiothreitol (DTT, dissolved in 50 mM ammonium bicarbonate). 10 μL of 150 mM iodoacetamide (dissolved in 50 mM ammonium bicarbonate) was then added to the sample and incubated at room temperature in the dark for 30 min. Fifteen microlitre (15 μl) of DTT was added to quench the alkylation. Two hundred micrograms (200 μg) of Sera-Mag SpeedBead carboxylate-modified magnetic particles (GE Healthcare, United States) were added to the sample, followed by pure acetonitrile (ACN) to give a final concentration of 70% ACN. The sample was incubated at room temperature for 10 min to allow protein binding. Magnetic particles were immobilised on the side of the tube by placing it on a magnetic rack to remove supernatant, followed by three washes with 80% ACN. Mass Spectrometry grade trypsin/Lys-C mix (dissolved in 50 mM ammonium bicarbonate, Promega Corporation, United States) in a 1:20 trypsin/LysC:protein ratio was added to the sample and incubated at 37°C for 16 h. The supernatant was collected, and digested glycopeptides were dried and reconstituted in LCMS grade water for LC-MS analysis.

### LC-MS of Tryptic Digested Glycopeptides

Five micrograms (5 μg) of tryptic digested antibody were analysed on a BioAccord LC-MS system (Waters Corporation, United States). The system configuration includes an ACQUITY UPLC I-Class PLUS with an ACQUITY RDa detector (a compact time-of-flight mass detector) controlled by the compliance-ready UNIFI Scientific Information System software platform (Waters Corporation, United States). Samples were separated using an ACQUITY UPLC Peptide BEH C18 column (130 Å, 1.7 μm, 2.1 mm × 100 mm, Waters Corporation, United States) at 65°C and 250 μL/min, with a 60 min gradient from 1 to 40% of 0.1% formic acid in acetonitrile (mobile phase B). 0.1% formic acid in LCMS water was used as mobile phase A. The RDa mass detector was operated in positive electrospray ionisation full scan with fragmentation acquisition mode (MSe) at 2 Hz acquisition rate with a mass range of 400–2,000 m/z. The capillary voltage was set at 1.2 kV, cone voltage at 30 V, and the desolvation temperature was kept at 350°C. Leucine Enkephalin (Waters Corporation, United States) was used as the LockSpray compound for real-time mass correction.

### BioAccord LC-MS System Data Preprocessing

Data obtained from the BioAccord LC-MS system were exported as .uep file using the UNIFI Scientific Information System (Waters Corporation, United States). LCMS features from the data were extracted, deconvoluted, and exported as a .csv file listing each feature with its retention time, neutral mass, and intensity using Progenesis QI software (Waters Corporation, United States). As the smallest glycopeptide (EEQY***N***STYR or EEQF***N***STFR with HexNAc) has a neutral mass of 1359.594, data points with a neutral mass lower than that were removed from the list.

### GraphMS Analysis

Preprocessed .csv file was analysed using GraphMS software for glycopeptide identification as described previously (Choo et al., 2019). GraphMS plotted LC-MS features (neutral mass vs. retention time) into the nodes of a graph network. GraphMS’s algorithm then detected nodes that have a neutral mass difference that matched a predefined list of masses of Hex, HexNAc, Fuc, HexHexNAc, and NeuAc. Pairs of nodes were selected within a defined retention time window (30 s for Hex, HexNAc, Fuc, and NeuAc; 50 s for HexHexNAc; 500 s for NeuAc). These identified nodes with proximate retention time were clustered into subgraphs and connected by graph theoretic edges. Glycan composition and peptide of the reference node of each subgraph was manually assigned if it matches the modified “N-glycan 309 mammalian no sodium” database. The database was modified by adding the neutral mass of peptides to the neutral mass of glycans to form a list of glycopeptide neutral mass [for example, the neutral mass of EEQY***N***[+N4H3]STYR (2486.981)is the sum of the mass of EEQY***N***STYR (1188.505) and N4H3 (1298.476)], GraphMS then automatically identified the remaining nodes that are connected to the reference node by tracing the path of known monosaccharide mass additions or subtractions. Intensity of glycan and glycosylation attributes are presented as relative abundance in percentage [intensity of a glycan (or sum of glycan with attribute of interest)/sum of intensity x 100%]. The output of GraphMS consists of an excel spreadsheet that has a list of assigned glycans composition and intensities which corresponds to abundance.

### N-glycan Release and Labeling

N-glycan of antibodies were released and labeled using GlycoWorks RapiFluor-MS (RFMS) N-glycan kit (Waters Corporation, United States) according to the manufacturer’s protocol. Briefly, 15 μg of dried antibody was reconstituted in 22.8 μL of LCMS grade water and 6 μL of 5% RapiGest solution (Waters Corporation, United States). The solution was incubated at 95°C for 5 min to denature the antibody. N-glycans were released enzymatically by adding 1.2 μL (600 U) of Rapid PNGase F (Waters Corporation, United States) followed by 10 min incubation at 55°C. Released N-glycans were labeled with 12 μL of the RapiFluor-MS Reagent Solution (fluorescence label) at room temperature for 5 min. The solution was diluted in 358 μL of ACN, followed by isolation using a GlycoWorks HILIC μElution Plate (Waters Corporation, United States). Isolated released N-glycans were dried and reconstituted in 9 μL of LCMS grade water, 10 μL of DMF, and 21 μL of ACN sequentially for LC-MS analysis.

### LC-MS of Released N-glycans

Ten microlitres (10 μL) of reconstituted released N-glycans were injected into an ACQUITY H-Class UPLC (Waters Corporation, United States) coupled to a Xevo G2S QTof mass spectrometer (Waters Corporation, United States). Samples were separated using an ACQUITY UPLC Glycan BEH amide column (130 Å, 1.7 μm, 2.1 mm × 150 mm, Waters Corporation, United States) at 60°C and 400 μL/min, with a 40 min gradient from 25 to 49% of 50 mM Ammonium Formate (mobile phase A). 100% ACN was used as mobile phase B. RFMS-labelled glycans were excited at 265 nm and measured at 425 nm with an ACQUITY UPLC FLR detector (Waters Corporation, United States). The MS1 profile scans of m/z 400–2,000 were acquired using the Xevo G2S-QTof in positive mode with an acquisition rate of 1 Hz. The electrospray ionisation capillary voltage was set at 2.75 kV, cone voltage at 15 V, desolvation gas flow at 800 L/h, ion source temperature and desolvation temperature were kept at 120°C and 300°C, respectively. Glu1-fibrinopeptide B (Waters Corporation, United States) was used as the LockSpray compound for real-time mass correction. RapiFluor-MS Dextran Calibration ladder (Waters Corporation, United States) was also injected into LC-MS to calibrate the retention time of sample peaks. The retention times were normalised using the dextran calibration curve to Glucose Units (GU).

### N-glycans Assignment

Released N-glycans data were analysed using the UNIFI Scientific Information System. Fluorescence peaks were integrated manually using the UNIFI Scientific Information System and relative quantitation of peaks was obtained by area-under-curve measurements followed by normalisation to the total area. Glycan assignment was done by matching Glycan Unit (GU) and m/z of each peak to the relevant UNIFI library.

### Statistical Analyses

Data are presented as mean ± standard error of the mean. Statistical analyses were performed by Student’s t-test, one-way ANOVA, or two-way ANOVA with Bonferroni’s post hoc test using GraphPad Prism 8 (GraphPAD Software Inc., United States). Multiple comparisons were performed between IgG subclasses, antibody groups, or culture media in ANOVA tests. Data obtained from Xevo G2-S QTof-UNIFI workflow and BioAccord-GraphMS workflow were pooled (three per antibody group) and analysed using linear regression analysis. The criterion for significance was *p* < 0.05 (*p < 0.05; **p < 0.01; ***p < 0.001; ****p < 0.0001).

## Results and Discussion

### An Optimised Workflow for Glycopeptide Analysis of Antibody Molecules

**FIGURE 1:**
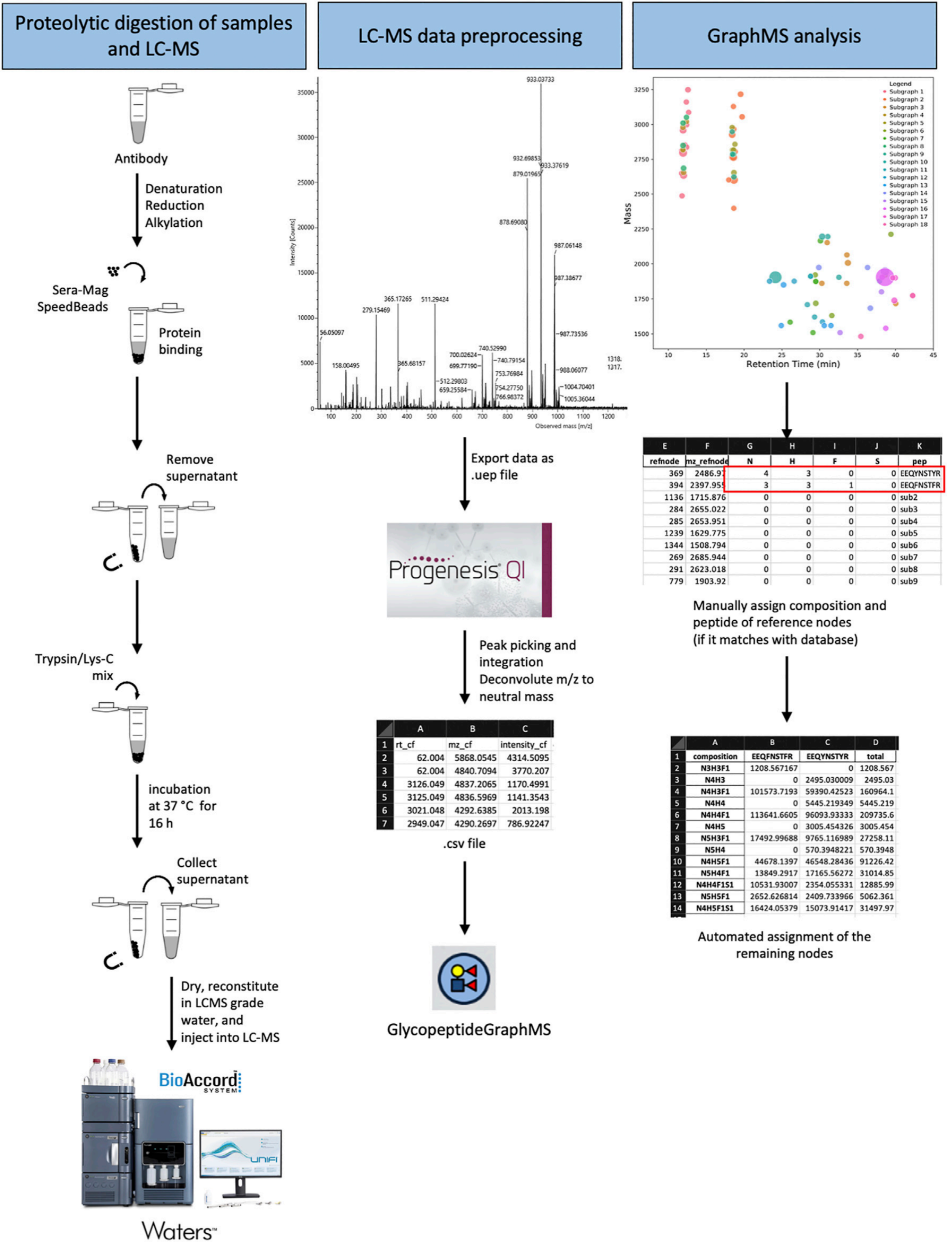
Schematic illustration of the glycoproteomic analysis workflow using GraphMS on Waters BioAccord LC-MS system data.

GraphMS is an algorithm that is designed to identify glycopeptide clusters with LC-MS data represented in a 2-Dimensional plot of mass and retention time (Choo et al., 2019). It does this by calculating the distance between nodes (of deconvoluted MS1 data) and clustering them within a certain retention time. Choo et al. (2019) have observed that glycopeptides from the same peptide backbone, with different glycan compositions, eluted at similar retention time. Hence, they are restrained by time and are clustered in the same subgraph (Choo et al., 2019). This step helps to extract potential glycopeptide nodes from the list of deconvoluted data which consists of background noise. To assess its usefulness in bioprocessing setting, we identified the glycopeptides from complex antibody-based datasets. We created datasets of trypsin/LysC digested IgG samples and biologics where there was minimal effort to enrich for glycopeptides as is the standard with a lot of glycopeptide/glycoproteomic protocols. Tryptic digested samples were loaded, separated, and analysed using the BioAccord LC-MS system using full scan with fragmentation mode (MSe). These protocols are schematically described in Figure 1.

With regards to analysis time, the assignment of glycan composition was significantly reduced with rapid and semi-automated assignment using a modified GraphMS workflow. Progenesis QI was used to deconvolute the accurate mass data while GraphMS was used to assign the glycopeptides. Without the need to cross-reference a database meant that there was little need to confirm the glycan assignments. The use of GraphMS in our glycoprofiling removed the over-reliance upon heavy user inspection and validation because glycan assignment with GraphMS was automatically validated by the presence of precursors and successive glycans in its clustered network. On average, data pre-processing with GraphMS workflow required less than 30 min to perform; this is significantly faster than released glycan manual assignment which takes approximately 1.5 h. The ease and semi-automatic analysis only require MS1 data and LC-MS data and is ideal for analysing large cohorts of data for lead or process development of antibody drugs as we demonstrate and assess their efficiency in different scenarios.

### Identification of Serum IgG Subclasses Glycoforms From Complex LC-MS Data Sets Using GraphMS

GraphMS was previously used to analyse LC-MS1 data acquired from Orbitrap Fusion Lumos MS coupled with a nLC system to characterise glycopeptides of human plasma IgG (Lippold et al., 2020). However, the use of the software on BioAccord LC-MS system has not been explored. Hence, we use human serum IgG as a test case to investigate the capability of BioAccord LC-MS system in the identification of IgG subclasses based on their glycosylation with the help of GraphMS software. The aim being to develop a workflow that can be used by non-experts in the glycoanalytical field. Human serum IgG consists of four subclasses, namely IgG1, IgG2, IgG3, and IgG4 (Vidarsson et al., 2014), and neutral masses of the tryptic glycopeptides are different. In addition, glycan profile of IgG subclasses is also different (Chandler et al., 2019). In this study, we demonstrated the ability of GraphMS to identify different subclasses from a dataset of a glycopeptides and peptides from a tryptic digest of serum IgG.

**FIGURE 2:**
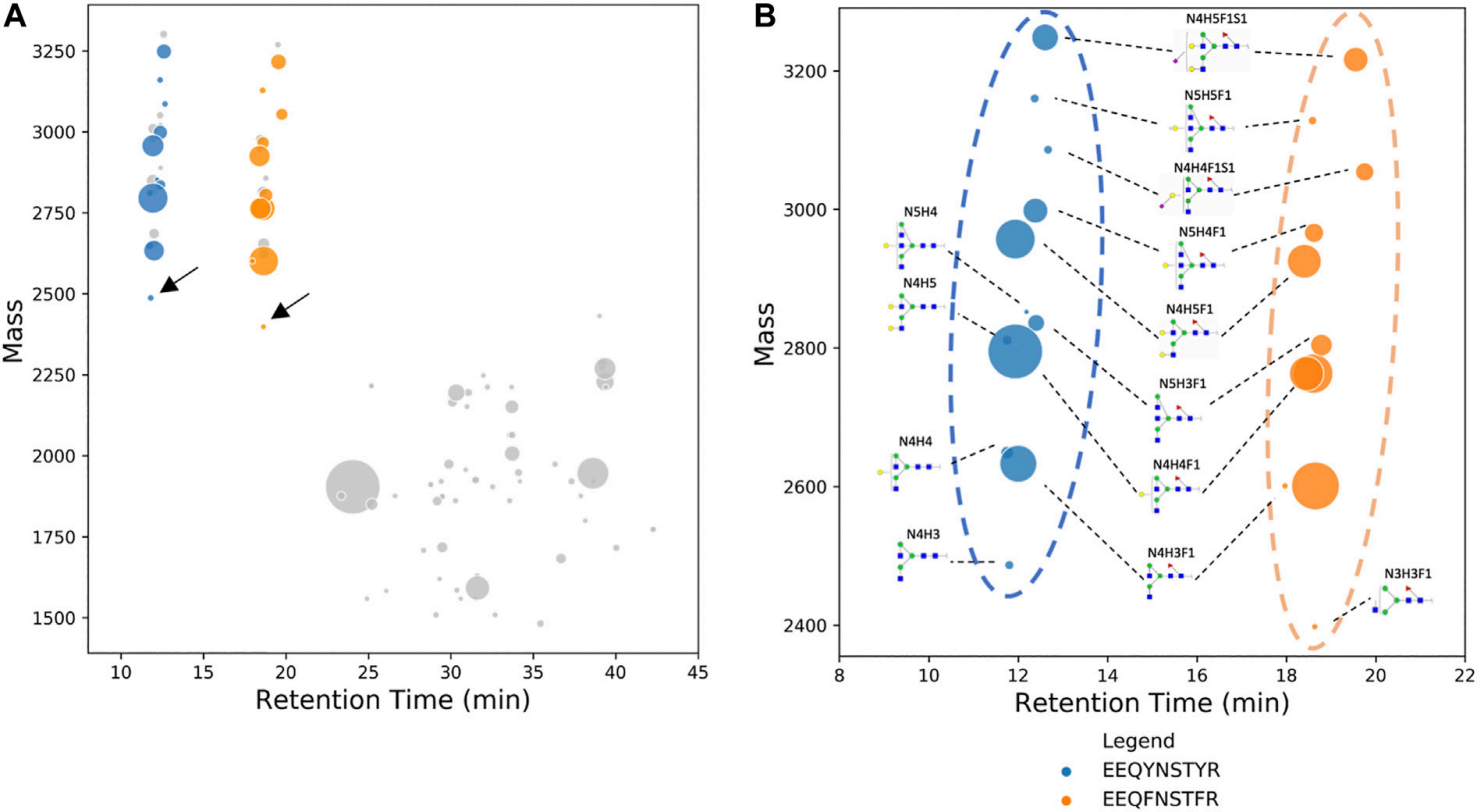
**(A)** Deconvoluted mass of human serum IgG glycopeptides are plotted on a bubble chart against their respective retention time. Nodes with the lowest neutral mass in each subgraph, also known as reference nodes, are compared against the database of IgG glycopeptide masses. Two reference nodes (labeled with arrows), among other non-glycopeptide nodes (in gray), matched IgG glycopeptide mass and their composition was manually assigned. **(B)** Bubble chart of the deconvoluted mass of glycoforms of EEQY***N***STYR (human serum IgG1) and EEQY***N***STYR (human serum IgG2) against their respective retention time. Identification of glycan composition was performed using GraphMS. The plot shows a clear distinction between IgG1 and IgG2. In some cases, glycopeptides are plotted separately as two nodes due to the slight difference in retention time (N4H3F1 and N4H4F1 of EEQY***N***STYR).

Feature nodes of human serum IgG were plotted on a bubble chart as shown in Figure 2A. From the tryptic digested serum IgG, we were able to identify a total of 18 subgraphs or clusters (Figure 2A). Out of 18 subgraphs, only reference nodes (labeled with arrows in Figure 2A) of 2 subgraphs (blue and orange color) matched the neutral mass of the modified database. Subgraphs with reference node that did not match the modified “N-glycan 309 mammalian no sodium” database (grey color, Figure 2A) were removed from the subsequent analysis. The two reference nodes that matched the modified database are EEQY*N*[+N4H3]STYR (IgG1, neutral mass 2486.981) glycopeptide and EEQF*N*[+N3H3F1]STFR (IgG2, neutral mass 2429.959) glycopeptide of human serum IgG. Glycopeptides EEQY*N*STYR (IgG1) and EEQF*N*STFR (IgG2) can be separated well as demonstrated in Figure 2B and they eluted at approximately 12 and 18 min, respectively; this is due to the difference in their hydrophobicity of the peptide backbone (Meek, 1980). Two distinct clusters can be identified and differentiated visually from dataset of glycopeptides and peptides.

**FIGURE 3:**
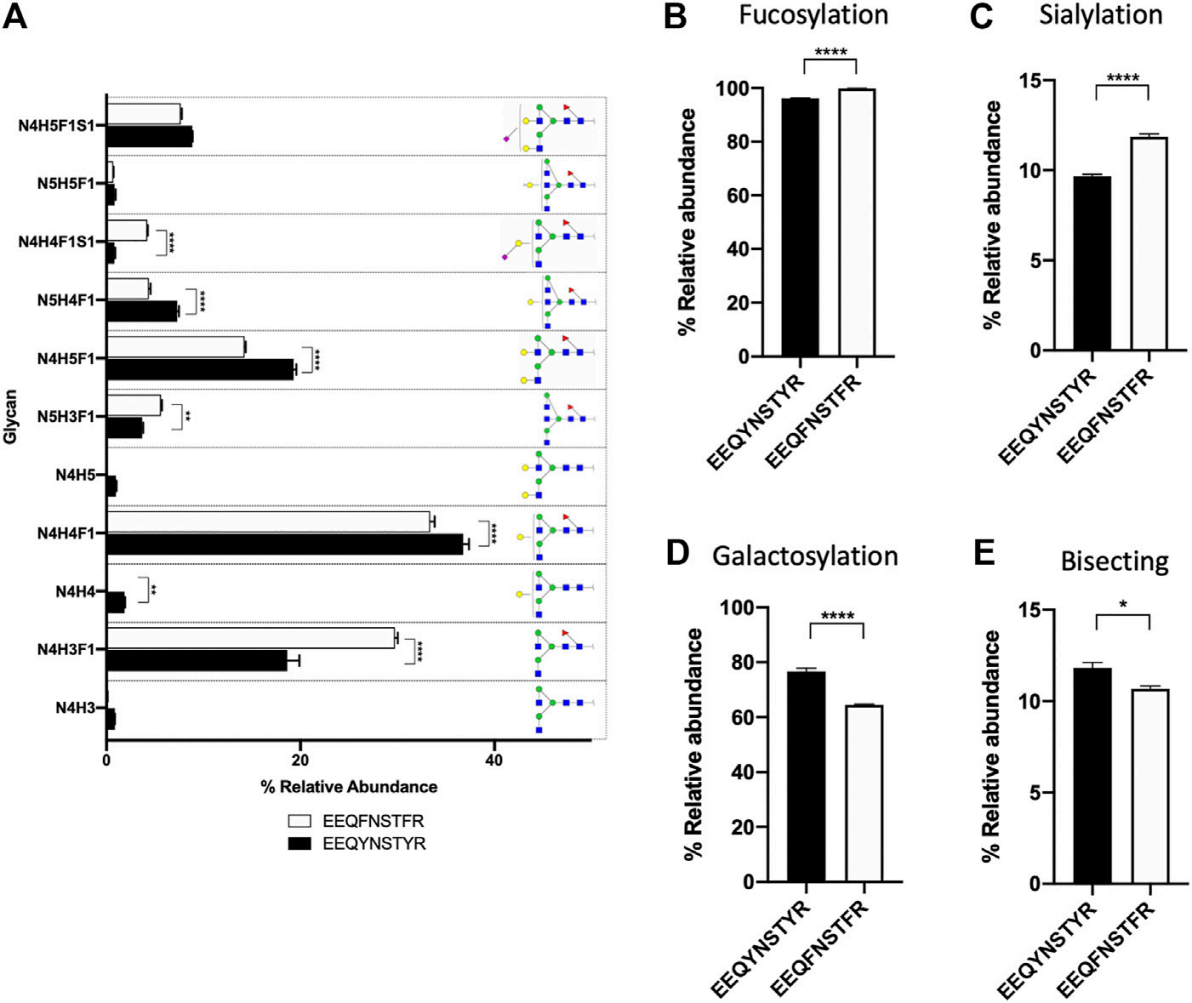
**(A)** Bar chart summarises the relative abundance (in percentage) of glycans of EEQY***N***STYR and EEQY***N***STYR (only those with relative abundance higher than 0.5% are plotted). Relative abundance of **(B)** fucosylation, **(C)** sialylation, **(D)** galactosylation, and **(E)** bisecting structure of serum IgG. **(A)**, Two-way ANOVA followed by Bonferroni’s post hoc test, multiple comparisons were performed between EEQY***N***STYR and EEQY***N***STYR, *n* = 4; **(B–E)**, Student’s t-test; *n* = 4, *p < 0.05, **p < 0.01; ****p < 0.0001).

Remaining nodes in each subgraph were identified automatically and without referencing any glycan or peptide database, as discussed in the previous section, and their glycan compositions are annotated in Figure 2B. In addition to composition assignment, GraphMS also plots intensity on the third dimension- bubble size, which corresponds to the abundance of the molecules. Thus, the visualisation shown in Figure 2B is a new way to present and quickly compare two IgG subclasses qualitatively and quantitatively. The relative abundance of each glycan on EEQY*N*STYR (IgG1) and EEQF*N*STFR (IgG2) peptides is summarised in Figure 3A (only those with relative abundance higher than 0.5% are plotted).

The relative abundance of seven out of 11 glycans of EEQY***N***STYR (IgG1) and EEQF***N***STFR (IgG2) peptides was found to be significantly different. For example, relative abundance of two most abundant glycans- N4H4F1 and N4H5F1, on EEQY*N*STYR (IgG1) were significantly higher than EEQF*N*STFR (IgG2) (Figure 3A, 36.782 ± 0.594% versus 33.368 ± 0.471%, *p* < 0.0001, and 19.284 ± 0.291% versus 14.222 ± 0.129%, *p* < 0.0001, respectively). Presence and abundance of IgG1 glycoforms analysed using workflow presented here are consistent with published data (Chandler et al., 2019; Cao et al., 2020). Glycosylation attributes, fucosylation, sialylation, galactosylation, and bisecting structure, of IgG1 and IgG2 were found to be significantly different (Figures 3B–E, *p* < 0.0001, *p* < 0.0001, *p* < 0.0001, and *p* < 0.05, respectively). It is, however, worth noting that only IgG1 and IgG2 are identified using BioAccord-GraphMS workflow. This might be due to the low relative abundance of IgG3 (4%) and IgG4 (4%) as compared to IgG1 (60%) and IgG2 (32%) and this study has not attempted to optimise the sensitivity of the workflow to capture that (Vidarsson et al., 2014).

### Identification of Trastuzumab and Adalimumab Glycoforms Using GraphMS

Trastuzumab (anti-HER2) and adalimumab (anti-TNF-α) are both humanised monoclonal IgG1 antibodies that target receptor tyrosine-protein kinase erbB02 (HER2) and tumor necrosis factor-alpha (TNF-α), respectively (Bang and Keating, 2004; Hudis, 2007). Although methods to analyse trastuzumab and adalimumab are well established (Yang and Bartlett, 2019), our workflow offers several advantages compared to traditional approaches- the BioAccord LC-MS system is simple operate by non-experts, the semi-automated GraphMS analysis was proven to be much faster (see results above), and the graphical visualisation of the glycopeptide identities and quantities allow comparison of multiple glycoproteins.

**FIGURE 4:**
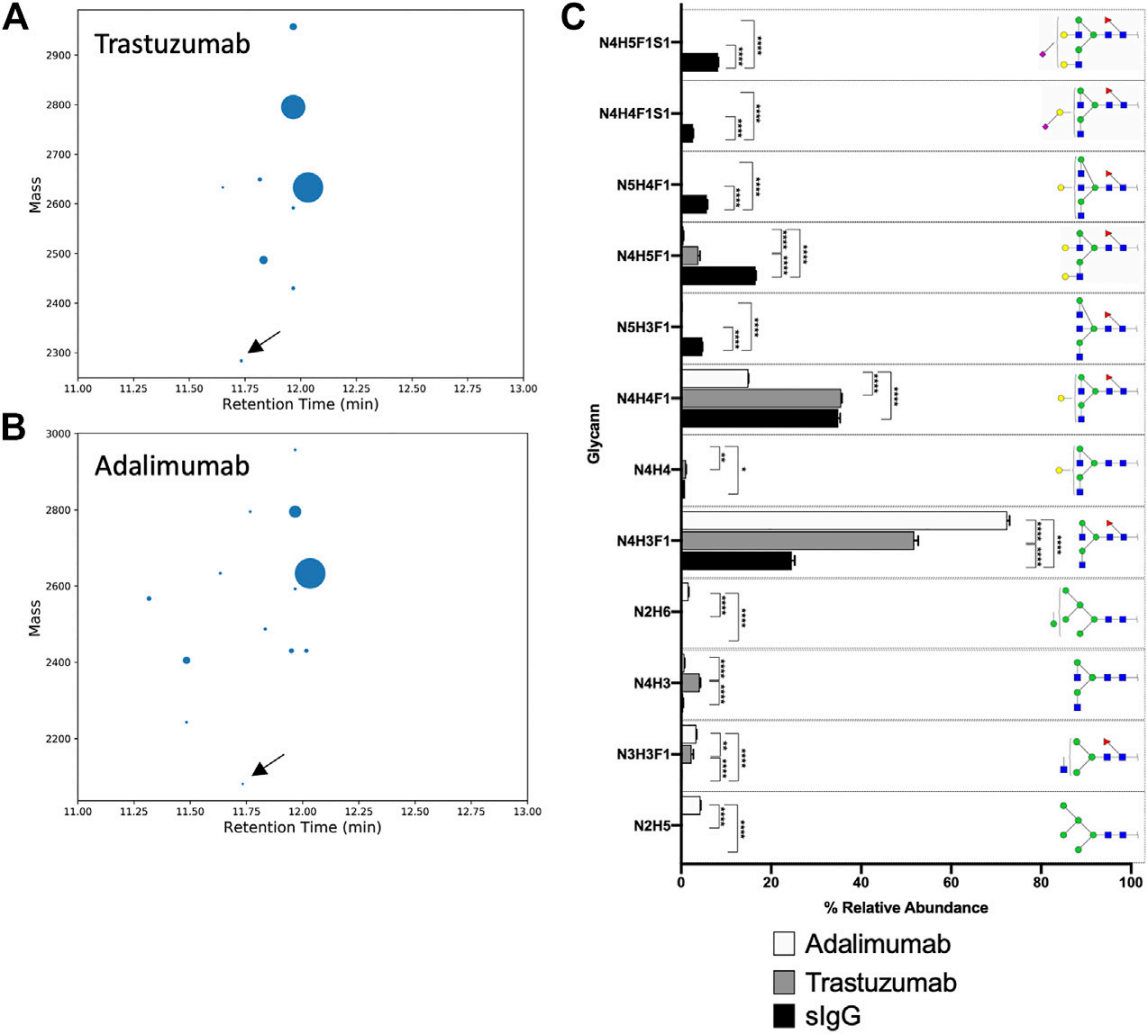
Bubble chart of the deconvoluted mass of glycoforms of EEQY***N***STYR of **(A)** trastuzumab, and **(B)** adalimumab, against their respective retention time. Identification of glycan composition was performed using GraphMS. Reference nodes of trastuzumab and adalimumab are labeled with black arrows. **(C)** Bar chart summarises the relative abundance (in percentage) of glycans of serum IgG, trastuzumab, and adalimumab (only those with relative abundance higher than 0.5% are plotted). **(C)**, Two-way ANOVA followed by Borreroni’s post hoc test, multiple comparisons were performed between serum IgG, transtuzumab, and adalimumab, *n* = 4; *p < 0.05, **p < 0.01; ***p < 0.001; ****p < 0.0001).

We demonstrate the use of BioAccord-GraphMS workflow to distinguish glycopeptides from the same IgG subtype of antibody molecules thus highlighting the effectiveness of the workflow to quickly characterise two typical biotherapeutic mAbs. The analysis can be easily extended to any number of mAbs used in the bioprocessing industry (e.g., for a large set of biosimilars). The GraphMS main output shows the neutral mass of tryptic digest of trastuzumab and adalimumab, and are plotted on bubble charts against their retention time as shown in Figures 4A,B. Both antibodies eluted at approximately 12 min, similar to that of human serum IgG1. Reference node of trastuzumab and adalimumab (labeled with arrows in Figures 4A,B) are EEQY*N* [+N3H3]STYR and EEQY*N* [+N2H3]STYR, respectively, and their compositions were manually assigned. GraphMS then automatically assigned the composition of the remaining nodes. The relative abundance of glycoforms of trastuzumab and adalimumab, together with those of human serum IgG, is summarised in Figure 4C (only those with relative abundance higher than 0.5% are plotted).

Differences in the glycoform of these antibodies were captured by our optimised glycopeptide analysis workflow. For instance, the relative abundance of the most abundant glycan- N4H3F1, of human serum IgG, trastuzumab, and adalimumab were found to be significantly different from each other (Figure 4C, *p* < 0.0001 for all comparisons). Bisecting and sialylated glycans (N5H3F1, N5H4F1, N4H4F1S1, and N4H5F1S1) were detected in human serum IgG and not in trastuzumab and adalimumab (Figure 4C, *p* < 0.0001 for comparisons of N5H3F1, N5H4F1, N4H4F1S1, and N4H5F1S1 between human serum IgG and trastuzumab or adalimumab).

**FIGURE 5:**
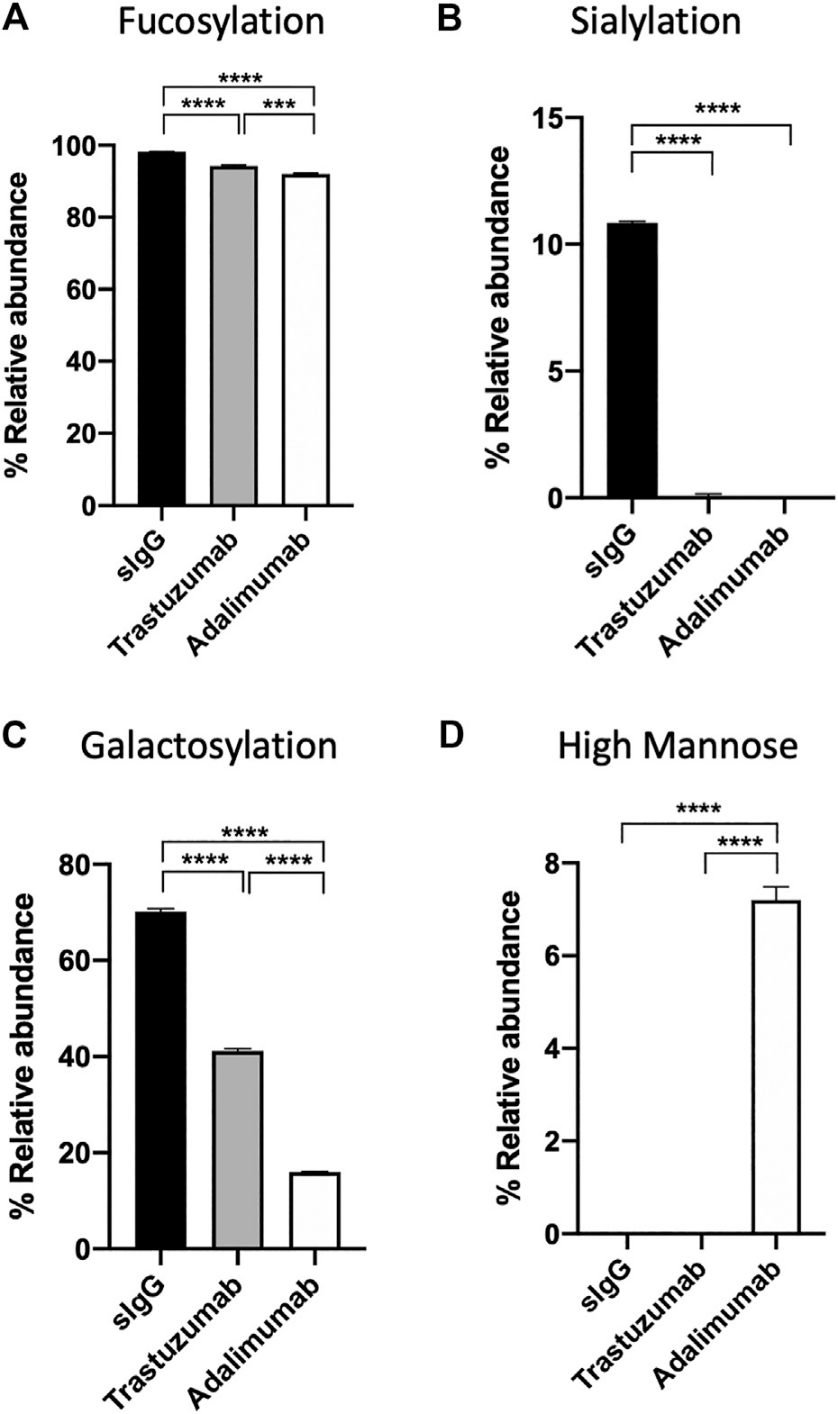
The relative abundance of **(A)** fucosylation, **(B)** sialylation, **(C)** galactosylation, and **(D)** high mannose structure of serum IgG, trastuzumab, and adalimumab. (One-way ANOVA followed by Bonferroni’s post hoc test, multiple comparisons were performed between serum IgG, transtuzumab, and adalimumab, *n* = 4; ***p < 0.001; ****p < 0.0001).

Amongst the three antibodies, human serum IgG was found to have a significantly higher relative abundance of fucosylated, sialylated, and galactosylated glycans than trastuzumab and adalimumab (Figure 5A–C, *p* < 0.0001 for all comparison between human serum IgG and trastuzumab or adalimumab). On the other hand, adalimumab was found to have significantly higher high mannose structure than human serum IgG and trastuzumab (Figure 5D, *p* < 0.0001 for all comparison between adalimumab and human serum IgG or trastuzumab). Despite the absence of MS2 data, the relative abundance and glycoforms of trastuzumab and adalimumab and their glycosylation profiles presented in this study are consistent with the studies often performed using techniques that require significant invested time and expertise to analyse LC, MS and MS2 data (Bandyopadhyay et al., 2015; Sanchez-De Melo et al., 2015). In contrast to that, the analysis of BioAccord-GraphMS workflow allow visualisation and annotation of glycan profile in less than 30 min.

### Comparison of Glycopeptide Data Analysed Using GraphMS Workflow and Released Glycan Workflow

Conventionally, glycan characterisation is performed by fluorescently labeling released N-glycans with 2-aminobenzamide (2-AB), 2-aminoanthranilic acid (2-AA), or RFMS, they can then be detected using FLR detector (Bigge et al., 1995; Lauber et al., 2015). Labeling glycans with these fluorophores also improve MS ionisation efficiencies, allowing higher quality MS signals to be acquired. Furthermore, tagging of RFMS, amongst these fluorophores, to glycans allow precise relative quantitation using FLR signals as the glycan is stoichiometrically covalently linked to the fluorophores (Lauber et al., 2015). We set to compare relative abundance of glycans identified using GraphMS workflow to a conventional released N-glycan analytical workflow.

**FIGURE 6:**
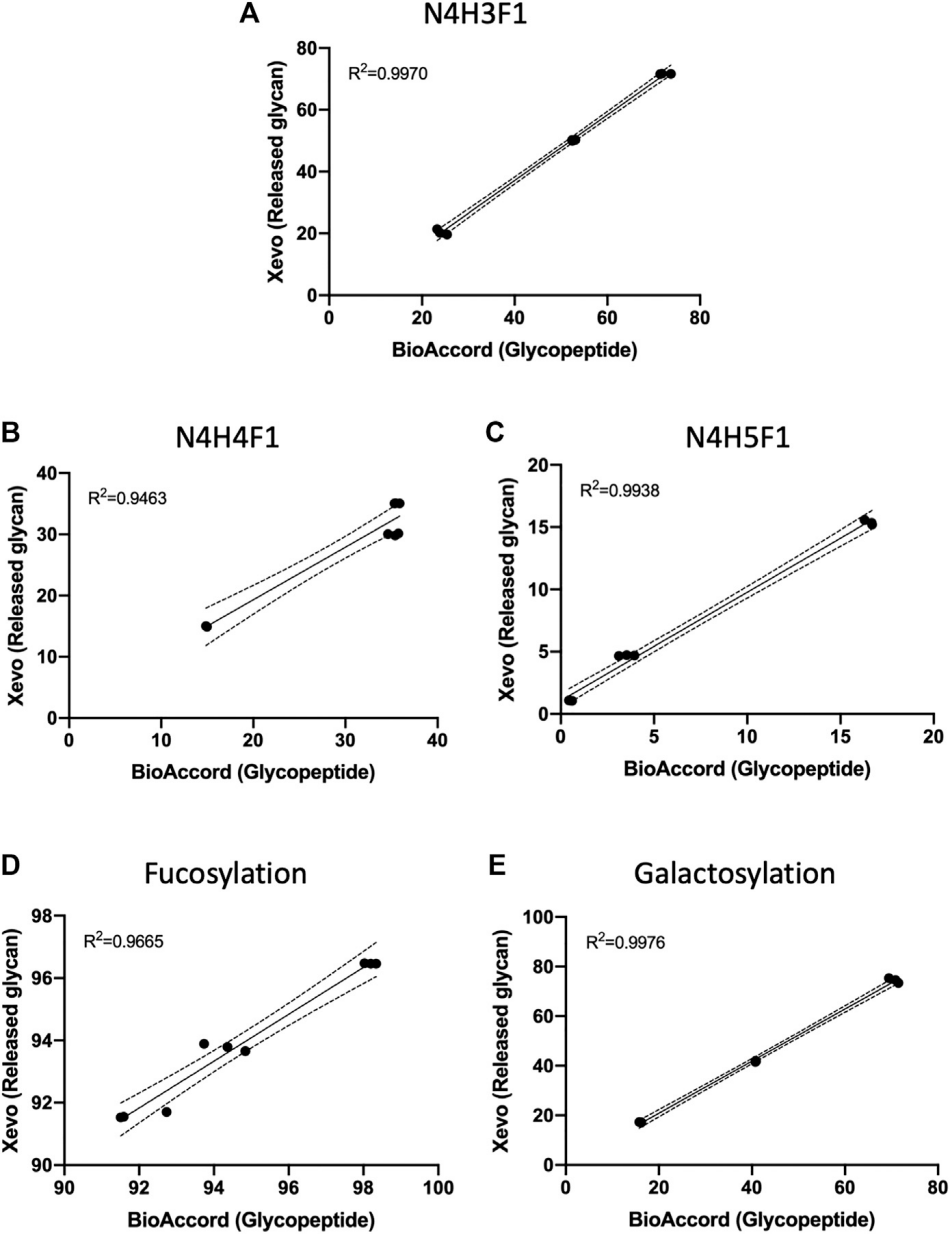
Data of the three most abundant glycans, **(A)** N4H3F1, **(B)** N4H4F1, and **(C)** N4H5F1, and glycosylation attributes, **(D)** fucosylation and **(E)** galactosylation, acquired and analysed using Xevo G2-S QTof workflow were highly correlated to BioAccord LC-MS system workflow. (Linear regression analysis, three replicates per antibody group, *n* = 9).

N-glycans of human serum IgG, trastuzumab, and adalimumab were chemoenzymatically released, fluorescently labeled and injected into Xevo G2S QToF mass spectrometer. Released N-glycans LC-MS data were analysed using the conventional method-manual assignment using UNIFI information system. Results obtained from the released N-glycans data were then compared with BioAccord-GraphMS workflow using linear regression analysis. From our results, there was high correlation between our LC-MS1 and GraphMS workflow to conventional released N-glycan analytical workflows. Figure 6 displays the relative abundance data of the three most abundant glycans- N4H3F1, N4H4F1, and N4H5F1, acquired and analysed using Xevo G2-S QTof-UNIFI workflow which was highly correlated to BioAccord-GraphMS workflow (Figures 6A–C, *R* ^2^ = 0.9970, 0.9463, and 0.9938, respectively). Similarly, glycosylation attributes like fucosylation and galactosylation were also found to be highly correlated (Figures 6D,E, *R* ^2^ = 0.9665, and 0.9976, respectively).

This demonstrates that the relative abundances of ion-intensity of a glycopeptide have high correlation to the stoichiometric abundance of glycans in both workflows. This comparison also supports the use of glycopeptide ion-intensity as a direct measure of glycosylation in multi-attribute monitoring (MAM) workflows that are emerging within the bio-analytical industry. The GraphMS data analysis workflow does semi-automatically generate this from collating the extracted ion intensities of the charge states of the candidate glycopeptide mass and combines this during the deconvolution stage (*via* Progenesis QI).

### GraphMS Data Analysis of Trastuzumab Biosimilar Fermentation Generates Longitudinal Characterisation of Bioprocess and Biologic Glycosylation

We set to demonstrate the utility of our described workflow in investigating the effect of different culturing conditions on the glycosylation of an in-house cultivated trastuzumab (anti-HER-2 antibody) biosimilar. This data is crucial to supplement studies and decision-making process in the cost, time efficiencies of production and importantly the safety profiles of the biologic. Hence, an efficient workflow like BioAccord-GraphMS allows the identification and quantitation would be useful when dealing with a multitude of data every other day of the bioprocessing operation. Trastuzumab antibodies were produced in either GE media or SAFC media in a fed-batch bioreactor and were harvested every 2nd day from days 3 to 14. Tryptic digested anti-HER-2 antibodies were injected into BioAccord LC-MS system and the LC-MS data were analysed using GraphMS.

**FIGURE 7:**
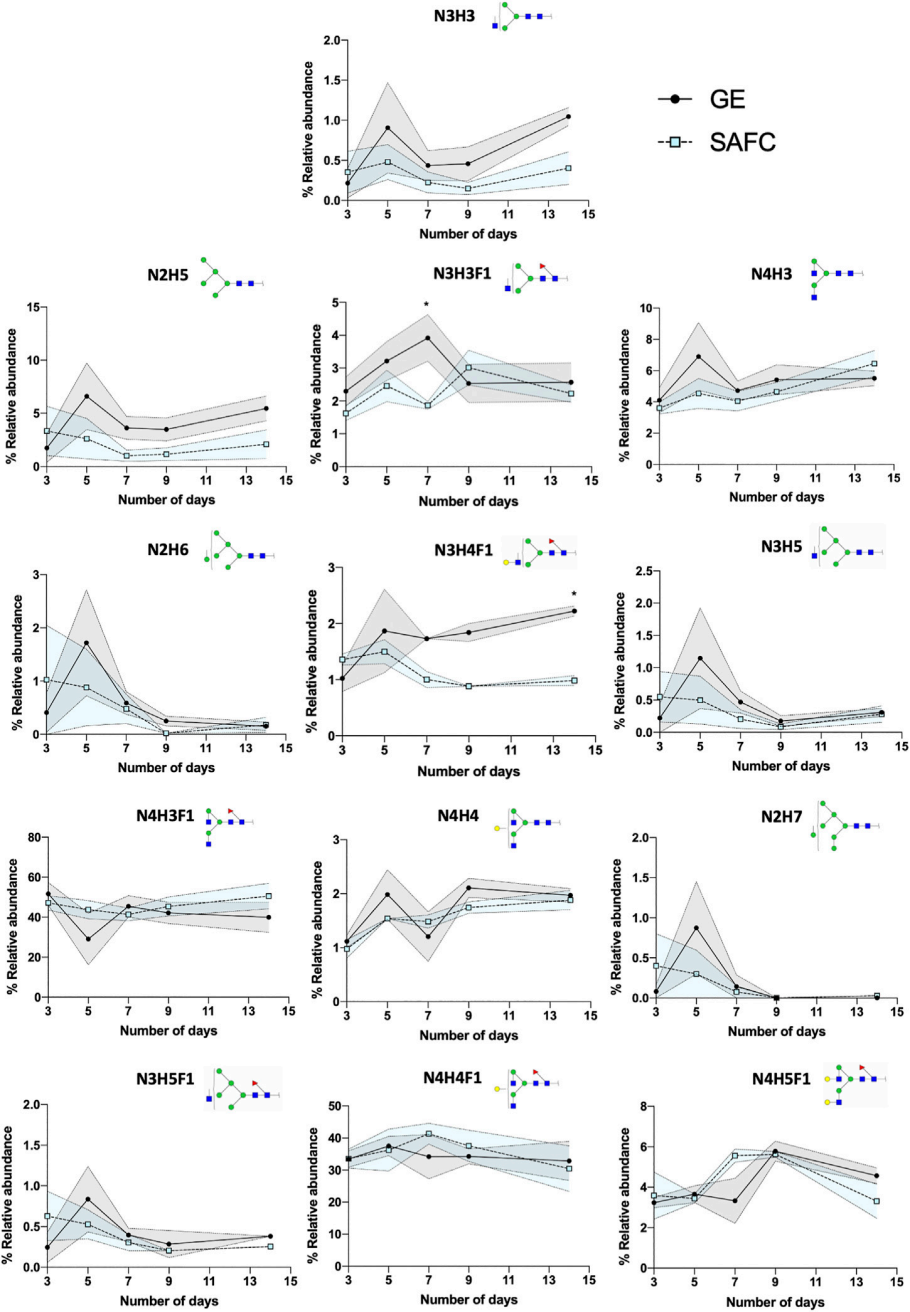
The relative abundance of in-house produced trastuzumab biosimilar glycoforms cultured in GE or SAFC media and on different length of duration. (Two-way ANOVA followed by Bonferroni’s post hoc test, multiple comparisons were performed between GE and SAFC media, *n* = 3; *p < 0.05; shaded area denotes ± 1 standard error).

The canonical antibody glycopeptide EEQY*N*STYR along with its resident glycans were identified, quantified, and monitored using GraphMS workflow. There were little differences in the abundance of the glycopeptides observed between the antibodies in different cultured media on the final day (day 14) of cultivation except for glycopeptide N3H3, N2H5, and N3H4F1 (Figure 7, 1.045 ± 0.112 versus 0.4028 ± 0.204, 5.454 ± 1.167 versus 2.093 ± 1.356, and 2.222 ± 0.091 versus 0.981 ± 0.088, respectively). Here, the glycosylation was increased in glycopeptides harvested in GE media compared to SAFC. Amongst the individual glycopeptides, the separation between GE and SAFC were most distinguished between N3H3F1 and N4H5F1 at around day 7 (Figure 7). These glycopeptides returned to comparable levels of glycopeptide abundance between the two media.

**FIGURE 8:**
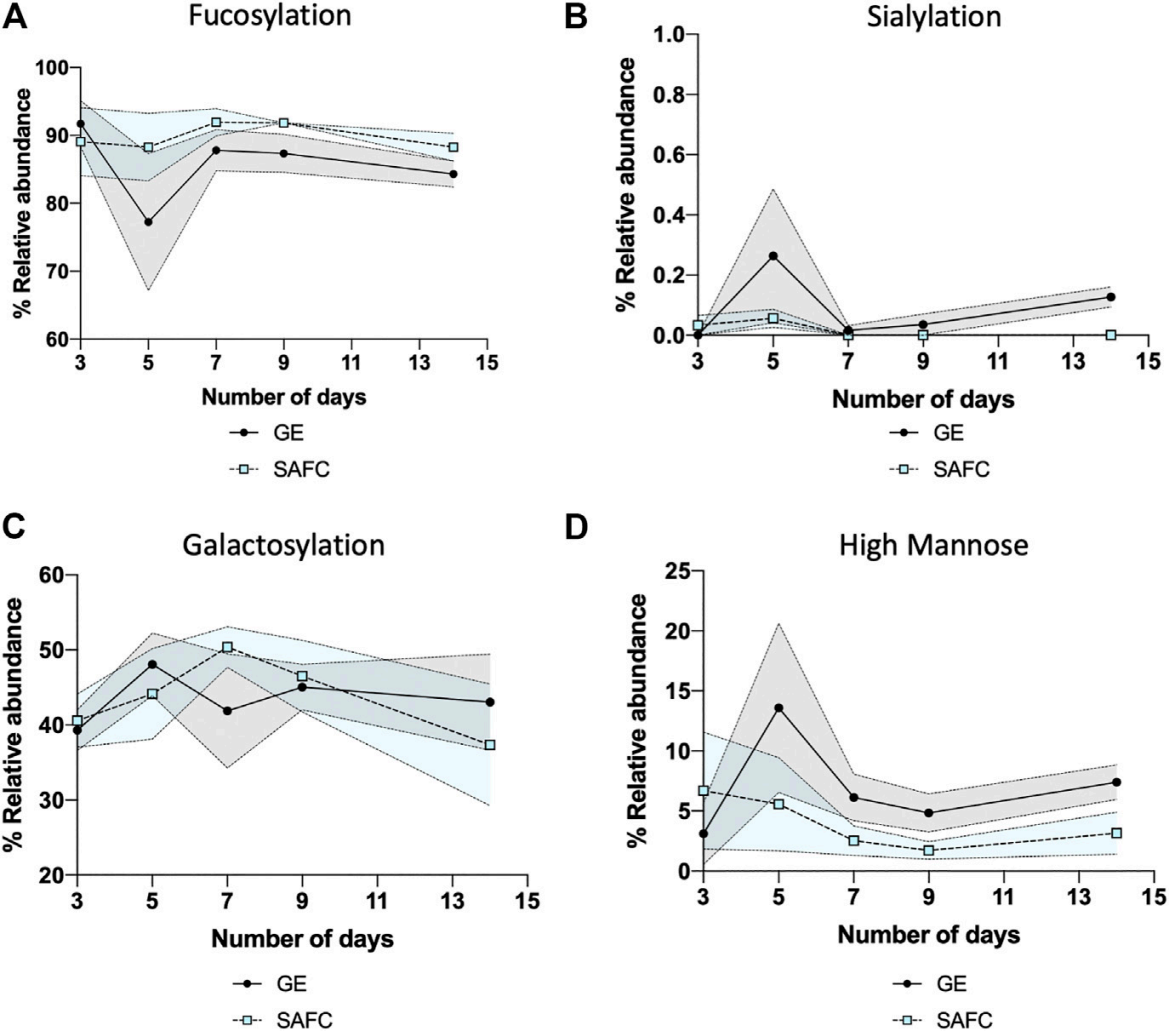
The relative abundance of **(A)** fucosylation, **(B)** sialylation, **(C)** galactosylation, and **(D)** high mannose structure of in-house produced trastuzumab biosimilar cultured in GE or SAFC media and on different length of duration. (Two-way ANOVA followed by Bonferroni’s post hoc test, multiple comparisons were performed between GE and SAFC media, *n* = 3; shaded area denotes ± 1 standard error).

In any large-scale bioprocess manufacturing, the possibilities for the abundance variations shown in Figures 7, 8 are vast. In general, glycan abundance changes in mAb bioprocessing can result from the cell expression systems used (Goh and Ng, 2018), CRISPR knock in/out of expression cell genes (Čaval et al., 2018), the culture and nutrient feed strategy used (Kildegaard et al., 2016), and physicochemical process parameters used (Ivarsson et al., 2014). In this study, our expression system and process parameters remained constant and therefore the differences seen in Figures 7, 8 between GE and SAFC are likely because of the varying quantities of the nutrients in the two media as well as contrasting initial stage and continuous feeding strategies in each media. Pinpointing the exact mechanisms that causes the glycan variation is beyond the scope of this article. To help find this mechanism a large longitudinal dataset will have to be generated that contains media nutrient identities and quantities, variation of process parameters recorded for each time point and the resulting glycopeptides identified and quantitated at each time point for each process parameter. For the latter, the number of glycopeptide analyses will be exceptionally large. Despite this, the BioAccord-GraphMS workflow described herein has shown promise in tackling this enormity as it can quickly report the identities and quantities of glycopeptides in our bioprocessing test case.

## Concluding Remarks

Analysis of glycosylation of an antibody is important because it directly affects the efficacy of the biotherapeutic. Glyco-analytics is quite challenging because it requires significant investment in expertise and infrastructure to adeptly characterise the glycosylation aspect of antibodies. Conventionally, glycosylation is analysed using one LC-MS or CE assay after the chemoenzymatic glycan release and conjugation of these glycans with a fluorescent label. The attraction behind this technique is that the quantitation is a direct stoichiometric abundance of the individual glycans. Within the biopharmaceutical development industry, multi-attribute monitoring (MAM) is an emerging and popular method for antibody development analytics because it allows for the relative quantitation and characterisation of antibody features and post-translational modifications of the peptides including but not limited to glycosylation, disulphide mapping, phosphorylation, C-terminal lysine residue clipping and N-terminal pyroglutamic acid formation (Rogers et al., 2018). This attraction lies not only in its comprehensivity but also in the ability of the method to use simple LC-MS data; in most cases detection of MS1 information is sufficient.

The challenge behind MAM has always been expertise to ensure that there is no compromise over the accuracy of analysis and characterisation. Glycosylation for example requires significant time to check and confirm the structures. This is especially true for unexpected glycans and glycan compositions that are not a part of any database. Furthermore, using LC-MS1 data to confirm the identity of glycans and glycopeptides raises the prospect of false positives especially when mass is the most absolute confirmation of a glycan. GraphMS introduces another layer of information—the distance of each glycopeptide within a cluster of related glycopeptides to confirm the identity of the glycopeptide. False-positive matches that do not belong to the cluster can be removed. Our approach is semi-automated and requires the mere confirmation of a reference node. Once the composition of a reference node is established, automatic assignment of the entire glycopeptide cluster can be performed within an expedient time frame. This contrasts with conventional methods which would require manual inspection of the MS of each node.

In our study, we have demonstrated the high correlation between conventional released glycan analytical methodologies and glycopeptide analysis via GraphMS. The results of this comparison do agree with established studies comparing glycopeptide abundance and released glycan (Wang et al., 2017). We have demonstrated the use of GraphMS to profile and differentiate IgG1 and IgG2 by virtue of their unique glycosylation in human serum. Most importantly, the utility of GraphMS to semi-automate the filtering of glycopeptides along with the assignment and quantitation of the glycopeptides from datasets of peptides and glycopeptides introduces an ease method that can be used to investigate large cohort glycosylation antibody fermentation studies. Upon applying this to such studies, we were able to understand the subtle effects of culture media to the relative abundance of antibody glycosylation and most importantly establish a longitudinal process signature of the bioprocess and cultivation. Our approach described here would be a powerful approach to complement MAM-based analytical platforms within antibody development industries.

## Data Availability

Data supporting the findings of this study are available from the corresponding author on request. The mass spectrometry proteomics data have been deposited to the ProteomeXchange Consortium via the PRIDE partner repository with the dataset identifier PXD025299.
